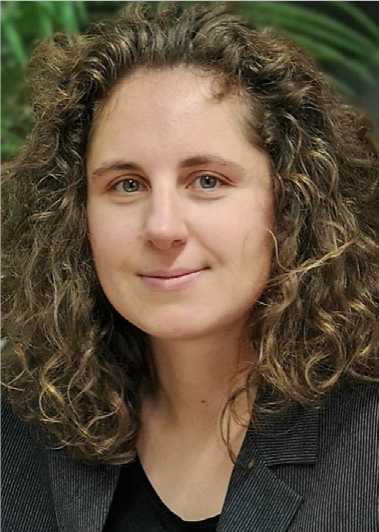# Catalysis with Palladium(I) Dimers

**DOI:** 10.1002/anie.202011825

**Published:** 2020-12-10

**Authors:** Christoph Fricke, Theresa Sperger, Marvin Mendel, Franziska Schoenebeck

**Affiliations:** ^1^ Institute of Organic Chemistry RWTH Aachen University Landoltweg 1 52074 Aachen Germany

**Keywords:** catalysis, cross-coupling, dinuclear Pd^I^, palladium, pre-catalyst

## Abstract

Dinuclear Pd^I^ complexes have found widespread applications as diverse catalysts for a multitude of transformations. Initially their ability to function as pre‐catalysts for low‐coordinated Pd^0^ species was harnessed in cross‐coupling. Such Pd^I^ dimers are inherently labile and relatively sensitive to oxygen. In recent years, more stable dinuclear Pd^I^−Pd^I^ frameworks, which feature bench‐stability and robustness towards nucleophiles as well as recoverability in reactions, were explored and shown to trigger privileged reactivities via dinuclear catalysis. This includes the predictable and substrate‐independent, selective C−C and C−heteroatom bond formations of poly(pseudo)halogenated arenes as well as couplings of arenes with relatively weak nucleophiles, which would not engage in Pd^0^/Pd^II^ catalysis. This Minireview highlights the use of dinuclear Pd^I^ complexes as both pre‐catalysts for the formation of highly active Pd^0^ and Pd^II^−H species as well as direct dinuclear catalysts. Focus is set on the mechanistic intricacies, the speciation and the impacts on reactivity.

## Graphical abstract

While dinuclear Pd^I^ complexes first received attention for being excellent pre‐catalysts that lead to high cross‐coupling reactivities, more recent work has uncovered that direct dinuclear catalysis can also take place and reaction modes are accessible which are not amenable to Pd^0^/Pd^II^ cycles. This Minireview highlights the use of dinuclear Pd^I^ complexes in catalysis showcasing their unique reactivity and selectivity.

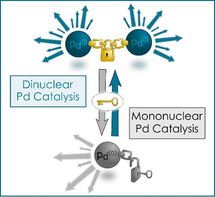

## 1 Introduction

Although the use of multiple metal centers to accomplish selective and efficient catalytic transformations is ubiquitous in nature's enzymes, most of homogeneous metal catalysis relies on the use of solely one metal center and only few multinuclear reactivity modes have been established.^[1]^ Despite the advantages of two or more metals working together to facilitate reactivity that might not be possible using either of the metals on their own, harnessing this synergistic power is not trivial due to the necessity of controlling nuclearity and speciation in solution. While nature uses protein backbones to control structure and speciation, in homogeneous metal catalysis similar controlling components are often absent as usually much smaller ligands are utilized. As a result, possibilities arise for different metal species to aggregate or dissociate, hence forming several potential active species with different nuclearity, ligation, and even oxidation state. In particular, owing to the effects of different additives, ligands, and the reaction conditions in general, relying on the distinct formation of just one active species to utilize its reactivity is challenging. Hence an understanding of the underlying processes that determine the fate of the different metal species and thus reactivity is of utmost importance.

In this context, palladium in its even oxidation states is omnipresent in homogeneous catalysis. By contrast, odd oxidation state palladium is comparatively scarce in catalysis. Due to their unpaired electrons and the potential formation of a rather strong Pd−Pd bond (usually about 25 kcal mol^−1^)^[2]^ these oxidation states tend to form dinuclear complexes. A wide variety of Pd^I^ dimers has been synthesized and has been known for decades. However, the utilization of dinuclear Pd^I^ in catalysis has only emerged in the early 2000s. In the search for enhancing the activity of Pd complexes used in catalytic applications, dinuclear Pd^I^ complexes were explored as pre‐catalysts that can release highly active Pd^0^ in situ. Truly dinuclear catalysis has been reported later in 2013 when our group provided unambiguous support for the direct involvement of a Pd^I^−Pd^I^ framework. With the advent of dinuclear Pd^I^ catalysis not only their unique reactivity and selectivity but also their straightforward application as robust, efficient, and reusable catalysts has been explored.

In this Minireview we (i) present the synthesis and properties of dinuclear Pd^I^ complexes relevant in catalysis, (ii) summarize their application as pre‐catalysts and the implications of their in situ formation, and (iii) showcase their utility in dinuclear catalysis.

## 2 Synthesis and Properties of Pd^I^ Dimers

**Figure 1:**
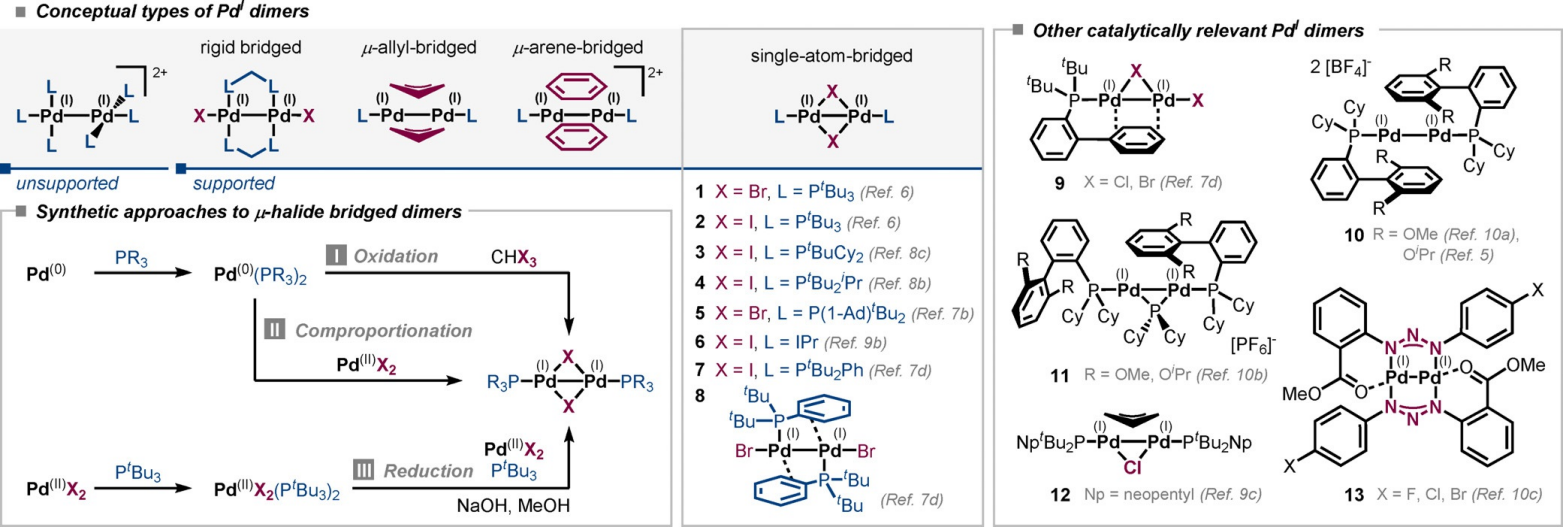
Conceptual types of Pd^I^ dimers, the general synthetic approaches to single‐atom‐bridged Pd^I^ dimers, and catalytically relevant examples (with reference to their first preparation). All structures were previously verified by X‐ray crystallographic analysis.

Pd^I^ dimers are connected by a Pd−Pd bond, forming the diamagnetic dimeric [Pd^I^−Pd^I^]^2+^ core unit while the single d^9^ Pd^I^ center possesses an unpaired electron. Pd^I^ dimers can be classified on the basis of their ligand structure into supported or unsupported dimers, depending on whether a bridging ligand is present or not. In unsupported dimers both Pd centers adopt a square planar geometry in which one coordination site is occupied by the other Pd atom forming a single Pd−Pd σ‐bond without the aid of any additional bridging ligands. In supported dimers the bonding between the two Pd^I^ centers is more extensive due to the influence of additional bridging ligands. Most commonly, Pd^I^ dimers are supported, that is, single‐atom‐ (e.g. halides), allyl‐, or arene‐bridged, as illustrated in Figure 1 (top). Furthermore, hybrids between the conceptual types of Pd^I^ dimers are feasible. For a detailed discussion of the structural diversity of Pd^I^ dimers we refer our readers to previous comprehensive review articles.^[3]^

Pd^I^ dimers are typically synthesized by (i) oxidation of Pd^0^,^[4]^ (ii) comproportionation of Pd^0^ and Pd^II^, or (iii) reduction of Pd^II^ (Figure 1). In addition to those direct approaches, Pd^I^ dimers can be accessed via ligand exchange from preformed Pd^I^ dimers.^[5]^

The first synthesis of μ‐halide‐supported dimers [Pd^I^(μ‐X)(P^*t*^Bu)_3_]_2_ **1** (X=Br) and **2** (X=I) was accomplished by oxidation of Pd^0^(P^*t*^Bu_3_)_2_ with highly activated electrophiles, for example, HCX_3_, *N*‐halosuccinimides or CX_4_ (X=Br or I).^[6]^ However, the desired Pd^I^ dimers could only be isolated in moderate yields and stoichiometric amounts of side‐products were generated.

Another strategy is the comproportionation of L_*n*_Pd^0^ and Pd^II^X_2_ (X=halide). This strategy was, for example, used in the synthesis of bromide‐bridged dimers **1**, **5**, and **9** from either PdBr_2_ or (cod)PdBr_2_,^[7]^ but also for Pd^I^ iodo dimers **2**, **3**, and **4** from PdI_2_.^[8]^ Usually the PdL_2_ complexes are preformed, but in case of dimer **2** it can also be formed in situ from commercially available Pd_2_(dba)_3_.^[8a]^

Furthermore, Pd^I^ dimers can be synthesized via reduction from corresponding Pd^II^ precursors, such as Pd^II^X_2_ or preformed mono‐ or dimeric Pd^II^ complexes. Reductive approaches were employed in the synthesis of Pd^I^ dimers **1**, **6**, and **12** with the aid of basic alkoxide solution,^[9]^ as well as in the synthesis of dimers **10**, **11**, and **13** where AgBF_4_, phosphine ligand, and Et_3_N served as key reagents and reductants.^[10]^

While attempts to synthesize the N‐heterocyclic carbene ligated dimer **6** by oxidation or comproportionation failed, the reduction from the corresponding Pd^II^ dimer [(IPr)Pd^II^I_2_]_2_ succeeded.^[9b]^ In contrast, the corresponding bromide‐bridged dimer remained elusive in an analogous synthesis attempt. The underlying reason is not well understood; however, reduction mechanisms to Pd^I^ can involve the formation of inorganic by‐products.^[9a]^

Moreover, there is a complex interplay of stabilizing ligands and anions to maintain the (+1) oxidation state in the Pd^I^−Pd^I^ core. For instance, the impact of the bridging halide is nicely illustrated in the formation of iodide‐bridged dimer **7**. While the Pd^I^ iodo dimer **7** was formed in high yield, the corresponding bromide‐bridged Pd^I^ dimer was not obtained under identical reaction conditions. Instead, its conformational isomer **8** possessing bridging arene units and ancillary bromide ligands is formed.^[7d]^ The precise effects of steric bulk and the donor–acceptor properties of the ancillary and bridging ligands on the structure, stability, and eventually the reactivity of Pd^I^ dimers are not yet understood and subject to ongoing research.

## 3 Applications in Cross‐Coupling

### 3.1 Pd^I^ Dimers as Pre‐Catalysts for Pd^0^

Although more than 50 different Pd^I^ dimers have been synthesized to date and characterized since the 1970s, only few were successfully implemented as pre‐catalysts in the ever‐growing field of metal‐catalyzed cross‐coupling.

**Figure 2:**
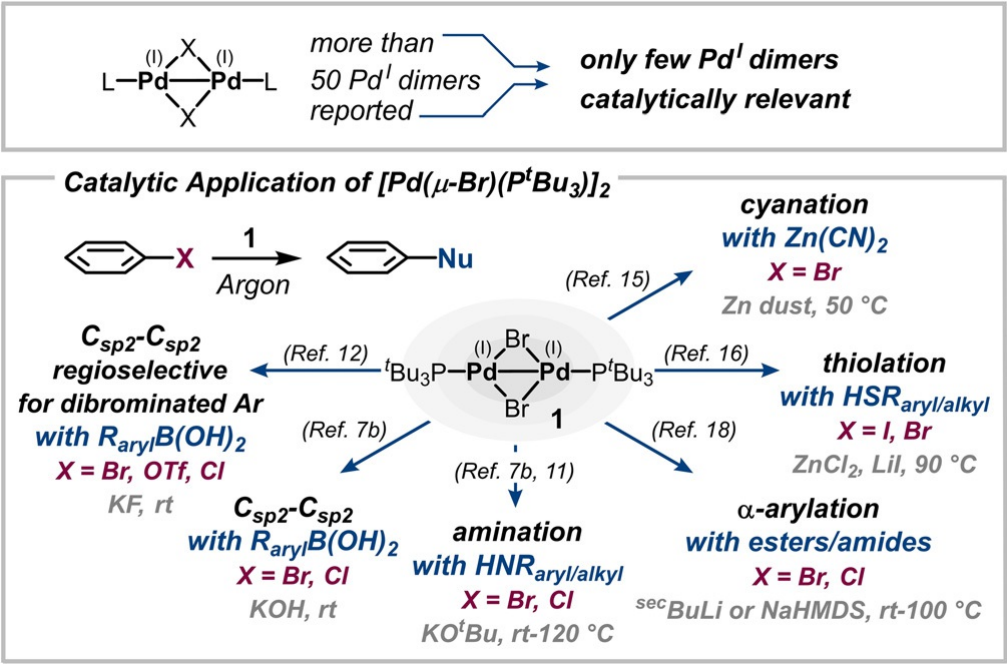
Application of Pd^I^ dimers as pre‐catalysts in Pd‐catalyzed cross‐coupling.[^7b^, ^11^, ^12^, ^14^, ^15^, ^16^, ^18a^, ^18c^, ^18d^, ^18e^, ^18f^]

In the early 2000s, Hartwig and researchers at Novartis independently demonstrated the synthetic potential of Pd^I^ dimers as highly reactive pre‐catalysts in Pd‐catalyzed amination reactions of aryl bromides or chlorides[^7b^, ^11^] and in Suzuki cross‐couplings of aryl bromides.^[7b]^ [Pd(μ‐Br)(P^*t*^Bu_3_)]_2_ (**1**) was used as pre‐catalyst, which was assumed (and later confirmed)^[12]^ to serve as an in situ reservoir for the highly reactive 12‐electron complex (^*t*^Bu_3_P)Pd^0^, which can readily activate aryl halides in catalytic transformations. A number of applications were subsequently demonstrated,^[13]^ such as C${}_{\mathrm{sp}{}^{2}}$ −NR (R=aromatic or aliphatic),[^7b^, ^11^, ^14^] C${}_{\mathrm{sp}{}^{2}}$ −C${}_{\mathrm{sp}{}^{2}}$ (Suzuki–Miyaura),[^7b^, ^12^] C${}_{\mathrm{sp}{}^{2}}$ −CN,^[15]^ C−SR (R=aromatic or aliphatic),^[16]^ or alkyne cross‐couplings^[17]^ and the α‐arylation/vinylation of carbonyl compounds^[18]^ (Figure 2).

Owing to its oxygen sensitivity, dimer **1** can only be handled for a very brief period in open laboratory conditions as a solid (e.g. for rapid weighing). In solution, it is essentially immediately deactivated in the presence of oxygen. In contrast, the iodinated dimer **2**[^7a^, ^19^] is completely stable in air as a solid and overall very robust. In fact, dimer **2** was unreactive under many of the conditions used for **1**, which might explain why, until the year 2013, there was only a single report of its application in catalysis which related to carbonylations of aryl halides.^[20]^

With the aim to improve the liberation of the catalytically active Pd^0^ species from Pd^I^ dimers, subsequent efforts focused on developing even more labile variants. In this context, Barder,^[10a]^ Vilar,^[7d]^ Spokoyny,^[5]^ as well as Shaughnessy and Colacot^[9c]^ independently established the use of Pd^I^ dimers **9**, **10**, and **12** as labile pre‐catalysts in Suzuki cross‐coupling, in amination, as well as in the α‐arylation of carbonyl derivatives.

The in situ release of the catalytically active species from the pre‐catalyst and its speciation influence the reactivity and selectivity in chemical transformations. In this context, the crucial question with regard to Pd^I^ dimers as pre‐catalysts was to identify the actual speciation, especially its nuclearity, to explain the distinct and frequently enhanced reactivity as compared to established Pd^0^ or Pd^II^ pre‐catalysts.

In 2012, our group examined the nature of active species derived from [Pd(μ‐Br)(P^*t*^Bu_3_)]_2_ (**1**) in Suzuki cross‐couplings of aryl bromides and chlorides, its potential activation mechanism, as well as the origins of limitations in scope.^[12]^ We confirmed that **1** solely serves as reservoir for the release of Pd^0^(P^*t*^Bu_3_)^[12]^ but is otherwise not reactive itself in these transformations. The high activity seen is due to a different activation mechanism of the complex as compared to, for example, L_2_Pd^0^ (L=P^*t*^Bu_3_), where dissociation of a phosphine ligand needs to occur which is associated with a significant energy penalty.^[21]^ By contrast, the Pd^I^ dimer **1** is most likely activated in a nucleophile‐assisted fragmentation.^[22]^ The direct in situ disproportionation (which had frequently been assumed in the literature prior to that) was ruled out on the basis of computational studies.

**Figure 3:**
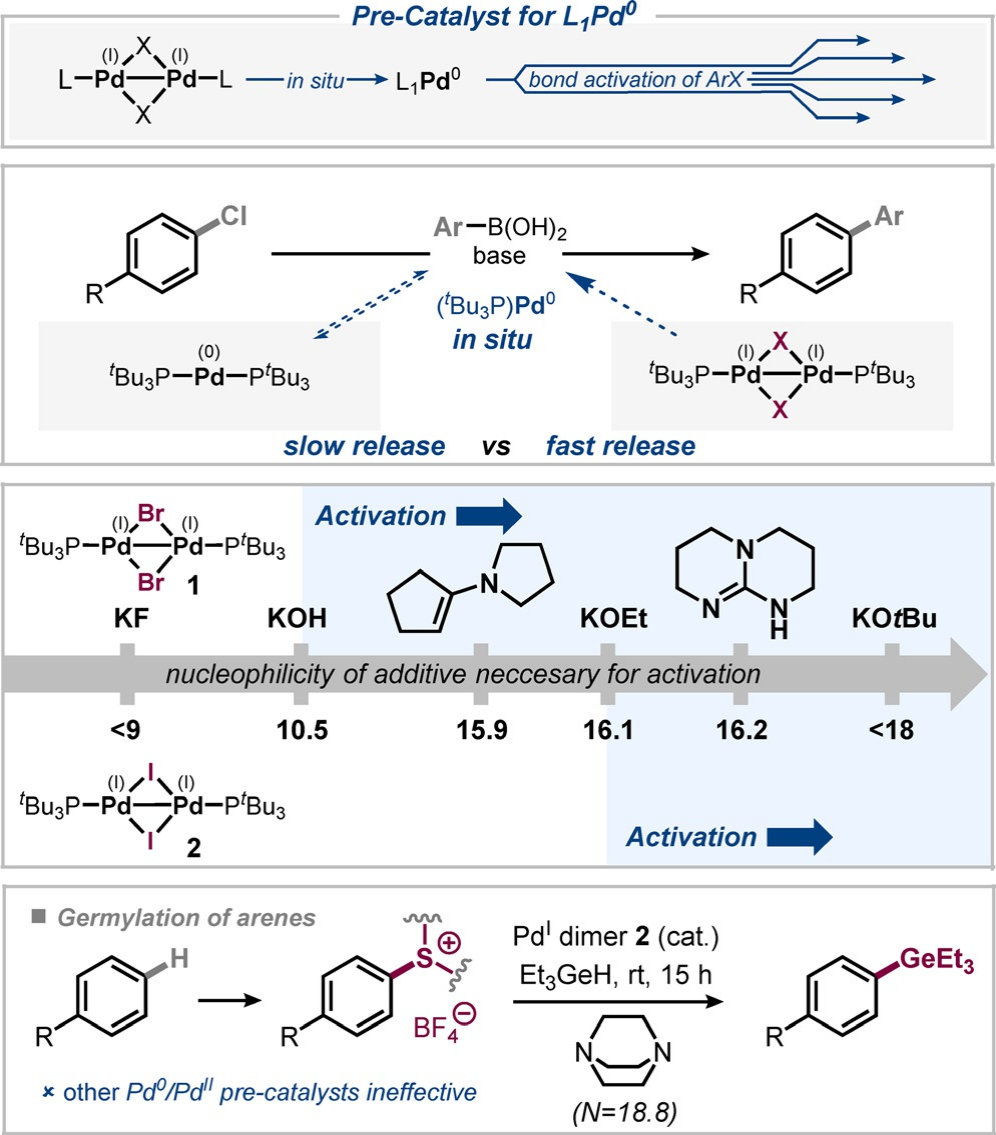
In situ liberation of catalytically active monoligated palladium from Pd^0^L_2_ or Pd^I^ dimer as pre‐catalysts (top)[^12^, ^21^] and *N*‐scale quantification for dimer activation (bottom).^[22]^ A suitable nucleophile is required for activation, which is present as a reagent or additive and should not function well as stabilizing bridge.

In Suzuki cross‐couplings, the activation of the Pd^I^ dimer (to Pd^0^) was found to be triggered by hydroxide or the mixture of boronic acid/KF, that is, frequently employed reagents and additives in Suzuki–Miyaura‐type cross‐couplings.^[22]^ By contrast, the corresponding iodide‐bridged Pd^I^ dimer, [Pd(μ‐I)(P^*t*^Bu_3_)]_2_ (**2**), is not activated by these species. Consequently, Pd^I^ dimer **2** does not catalyze Suzuki cross‐couplings with boronic acid/KF conditions. However, our group showed that the in situ release of Pd^0^(P^*t*^Bu_3_) from these Pd^I^ dimers is dependent on the adequate choice of additive,^[22]^ and have established the minimum nucleophilicity necessary (*N* scale)^[23]^ to do the activation in each case (Figure 3). Using oxygen‐ or nitrogen‐centered nucleophiles with *N*≥16 also activated Pd^I^ dimer **2** and with such bases, Suzuki coupling was then possible.^[22]^

Within our ongoing research program regarding the application of aryl germanes^[24]^ in synthesis and catalysis we found DABCO (*N*=18.80)^[25]^ to be a suitable nucleophile for the activation of Pd^I^ dimer **2** and subsequent functionalization of aryl tetrafluorothianthrenium salts (Figure 3).^[26]^ Thus, a mild and selective method was developed which allows the direct conversion of non‐activated aromatic C−H sites into aryl germanes via the tetrafluorothianthrenium salt as key intermediate.^[26b]^ It is worth noting that we observed that those pre‐catalysts, which had previously been reported to efficiently transform thianthrenium salts, did not give rise to efficient C−GeEt_3_ bond formation, nor did [Pd^0^(P^*t*^Bu_3_)]_2_.

### 3.2 In situ Formation of Pd^I^ Dimers and its Implications

Aryl bromides appear especially privileged in reactions with Pd^I^ dimers **1** or **2**. A detailed study of the Suzuki coupling of aryl chlorides with dimer **1** indicated that, although the coupling product forms relatively rapidly, a plateau in conversion is reached and hardly any further conversion to product takes place thereafter.^[12]^ The data indicated that ligand exchange from Pd^0^(P^*t*^Bu_3_) to form Pd^0^(P^*t*^Bu_3_)_2_ and Pd‐black (under precipitation of Pd) occurs over time. Once Pd^0^(P^*t*^Bu_3_)_2_ is solely remaining, reactivity is low. On the other hand, the complete conversions in cross‐couplings of aryl bromides with dimer **1** might potentially originate from an in situ regeneration of **1**. For example, Hartwig identified an unusual autocatalytic behavior in the oxidative addition to ArBr by P^*t*^Bu_3_‐derived Pd^0^ catalysts in which, among other species, the formation of Pd^I^ dimer **1** was detected. However, in this study the autocatalytic behavior was ultimately ascribed to (P^*t*^Bu_3_)_2_Pd^II^(H)(Br) being formed.^[27]^

**Figure 4:**
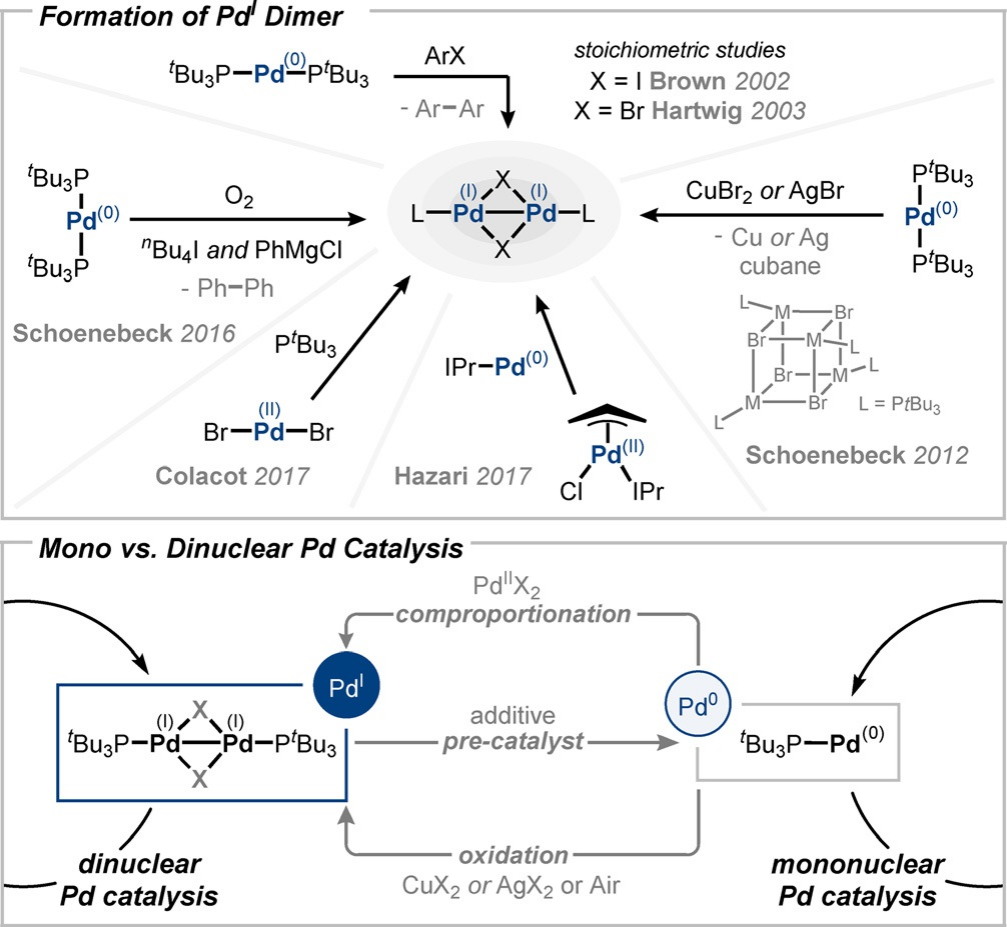
Generation of Pd^I^ dimer **1** under cross‐coupling conditions.[^9a^, ^27^, ^28^, ^29^]

As part of our investigations of cross‐couplings in air with Pd^I^ dimer **2** (discussed in Section 3.4) we discovered that Pd^I^ dimers can also be formed by aerobic oxidation of Pd^0^ species.^[28]^ When Pd^0^(P^*t*^Bu_3_)_2_ was treated with ^*n*^Bu_4_NI and PhMgCl (2 equiv relative to Pd) in the presence of oxygen, the corresponding Pd^I^ dimer **2** was formed along with biphenyl. These data indicated that any Pd^0^ species released in the reaction mixture during catalysis can, in principle, be transformed back to Pd^I^ under suitable conditions (Figure 4).

Colacot and his team found that the combination of phosphine ligand and Pd^II^Br_2_ gives rise to the formation of Pd^I^ dimer **1**, which was ultimately harnessed in a process for the synthesis of **1** on larger scale.[^9a^, ^29c^] In a collaborative experimental and computational study, we examined the mechanistic intricacies of this unusual reduction from Pd^II^ to Pd^I^ (as opposed to Pd^0^). It was found that unusual side species may also form in the process (such as (BrP^*t*^Bu_3_)(Br) and [Pd_2_^II^Br_6_][(BrP^*t*^Bu_3_)(Br)]_2_), which in turn are greatly influenced by the ligand stoichiometry, additives, or solvent. The implications for the catalytic performance were also illustrated.^[9a]^

In 2014, the group of Hazari observed the formation of Pd^I^ dimers by in situ comproportionation of (IPr)Pd^0^ and (IPr)Pd^II^(Cl)(allyl) complexes.^[29b]^ It was demonstrated that by modulating the steric demand of substituents on the allyl ligand an improved catalytic performance in cross‐couplings can be obtained. Increased steric bulk on the ligand was found to accelerate the release of Pd^0^ and decrease the tendency to (re‐)dimerize, overall favoring the in situ liberation of catalytically competent Pd^0^. These studies demonstrate the multitude of in situ activation and deactivation pathways for Pd (pre‐)catalysts via readily occurring redox processes and hence reinforce the challenges associated with such interchangeable Pd speciation in defining the catalytically active species in Pd catalysis.

In 2012, our group reported on the impact of additives on the in situ formation of Pd^I^ dimers and the resulting rate‐accelerating or inhibiting effects in catalysis.^[29a]^ We uncovered that upon addition of a Cu or a Ag salt—common additives in, for example, Sonogashira or Suzuki cross‐coupling reactions—to Pd^0^(P^*t*^Bu_3_)_2_ a Pd^I^ dimer and the corresponding Cu^I^ or Ag^I^ cubanes are formed. We showed that with CuBr_2_ the highly reactive [Pd(μ‐Br)(P^*t*^Bu_3_)]_2_ **1** is generated from Pd^0^(P^*t*^Bu_3_)_2_, which in turn can liberate Pd^0^(P^*t*^Bu_3_) more readily in situ and consequently accelerate diverse transformations. In stark contrast, the corresponding cross‐couplings with CuI are often slowed or even completely inhibited. We found that upon addition of CuI, [Pd(μ‐I)(P^*t*^Bu_3_)]_2_ **2** is formed in a redox process.^[29a]^ However, in comparison to Pd^I^ bromo dimer **1**, [Pd(μ‐I)(P^*t*^Bu_3_)]_2_ **2** displays high stability in solution towards diverse nucleophiles and is thus less competent to release Pd^0^(P^*t*^Bu_3_) as active species in C−C couplings, which in turn leads to decreased activity. Moreover, we found that Pd^I^ dimers **1** or **2** are competent to react directly with alkynes, and their in situ formation under typical Sonogashira coupling conditions (i.e., Cu salt/Pd^0^) will hence lead to an unproductive consumption of alkyne as competing process.^[29a]^ This side‐process is especially pronounced in the Sonogashira coupling of aryl chlorides and delivers an explanation of the previously observed inhibiting effects of Cu or Ag additives in Sonogashira couplings.^[30]^

### 3.3 Mechanistic Support for Dinuclear Reactivity

**Figure 5:**
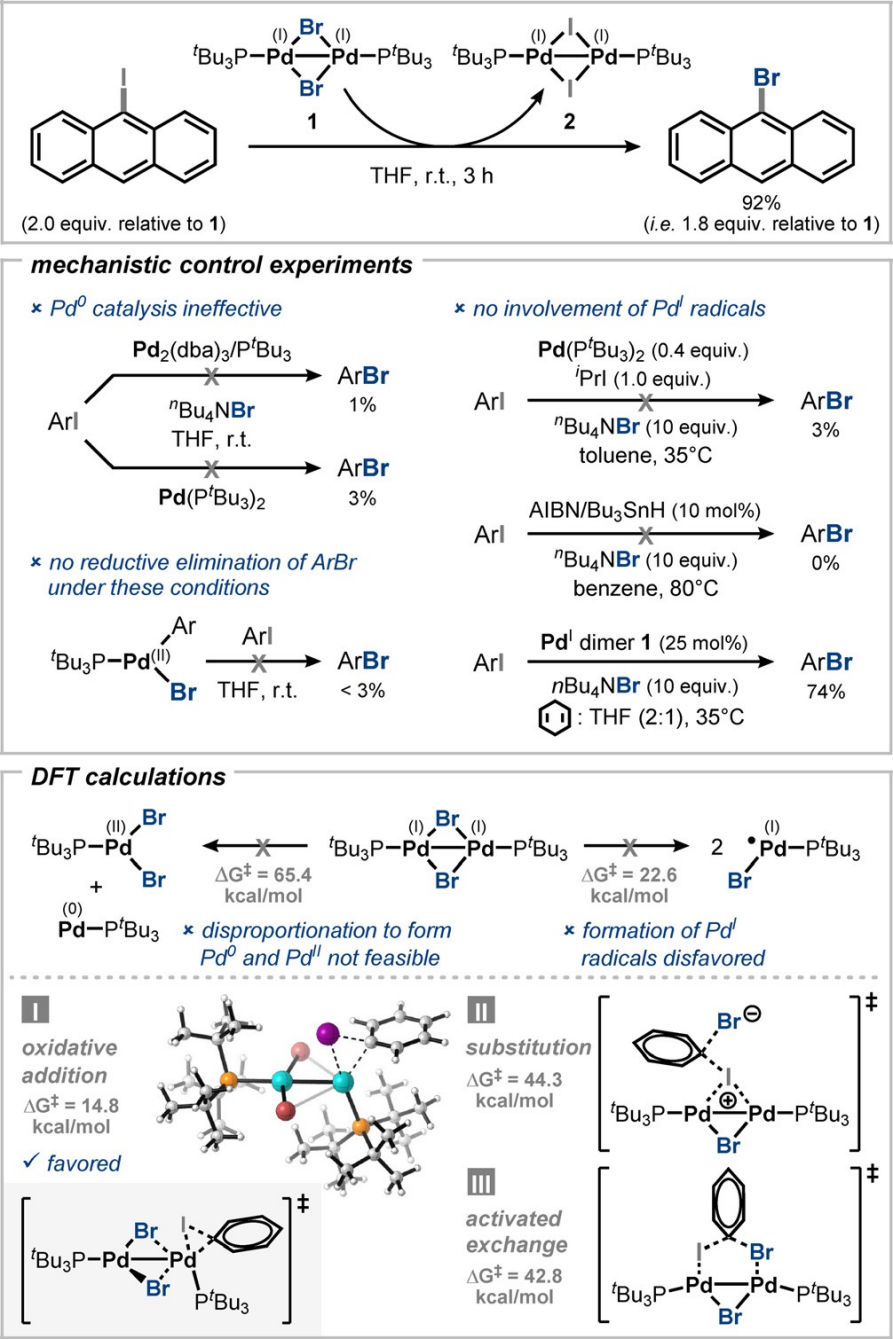
Dinuclear Pd^I^‐mediated halide exchange—experimental and computational support for dinuclear catalysis.[^31^, ^32^]

The catalytic role of Pd^I^ dimers was solely ascribed to being pre‐catalysts until the year 2013, when our group disclosed that Pd^I^ dimers could also react directly with an aryl halide. In a combined experimental and computational study we provided unambiguous support of this and provided a molecular mechanistic picture.^[31]^ We discovered that Pd^I^ dimer **1** can undergo a halide exchange reaction with aryl iodides to form aryl bromides by formally exchanging the μ‐bridging bromide for iodide (Figure 5). ^31^P NMR revealed the formation of different dimers, generated upon exchange of one or both of the bridges from bromide to iodide, but no species related to Pd^0^, Pd^II^, or free phosphine ligand were detected in the stoichiometric process. Moreover, Pd^0^ was shown to be ineffective to trigger this halogen exchange, and an independently synthesized Pd^II^ complex did not lead to formation of the product via reductive elimination, overall excluding the involvement of Pd^0^ or Pd^II^. A potential homolytic cleavage of the dimer into Pd^I^ radicals was found unlikely because the computed activation barrier is roughly 8 kcal mol^−1^ higher than the alternative direct oxidative addition at dinuclear Pd^I^. This is also in line with the fact that no paramagnetic species were observed in the course of the reaction. Additionally, when radical initiators such as AIBN/Bu_3_SnH were employed, no product formation was observed. Likewise, the addition of H‐atom donors such as 1,4‐cyclohexadiene did not significantly influence the reaction outcome.^[31]^ Kinetic studies furthermore corroborate dinuclear reactivity as first‐order dependence in Pd^I^ dimer was observed.^[32]^

Based on these observations as well as computational studies, a dinuclear mechanism was proposed. Several modes of activation of the aryl halide were explored computationally (Figure 5, bottom) including (i) oxidative addition, (ii) activated exchange at the dinuclear platform, and (iii) an *ipso*‐substitution where a cationic dimer acts as a Lewis acid. The oxidative addition mechanism was found to be clearly favored by almost 30 kcal mol^−1^ over the alternatives. Interestingly, while the calculated transition state suggests bond activation to occur primarily at one Pd center of the Pd^I^ dimer, after oxidative addition a Pd^II^ dimer was computationally obtained, which suggests that overall a Pd^I^−Pd^I^/Pd^II^−Pd^II^ oxidative addition occurs, which involves both Pd centers (instead of a Pd^I^/Pd^III^ mechanism).

**Figure 6:**
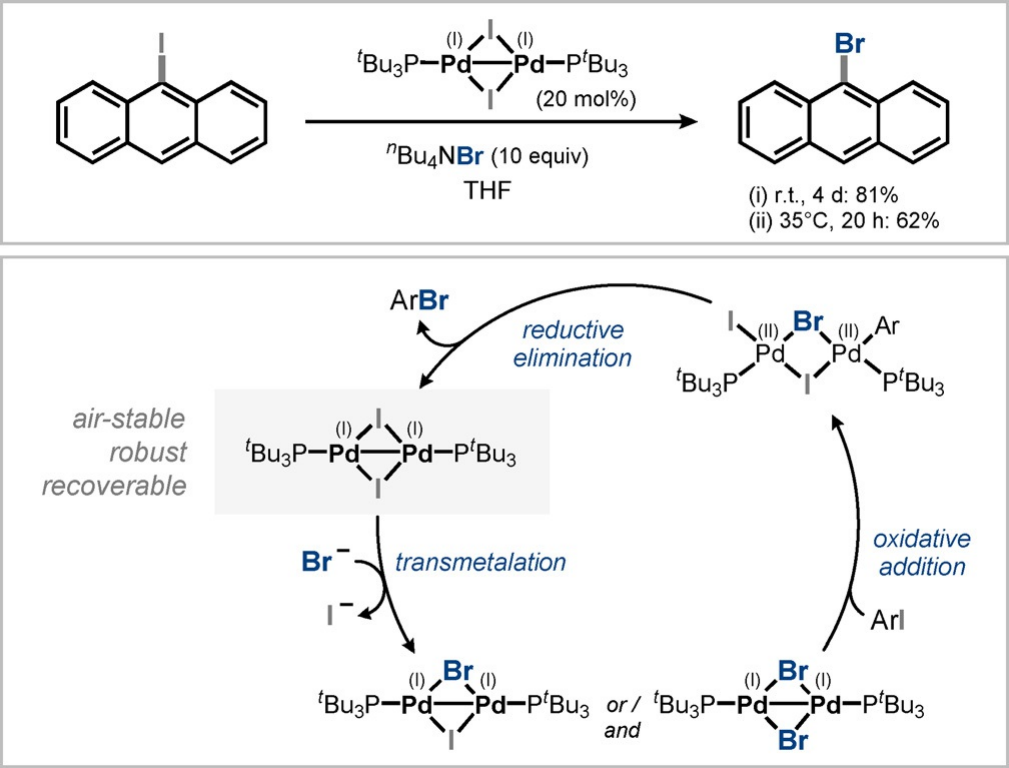
First dinuclear Pd^I^ catalysis (top) and proposed mechanism (bottom).[^31^, ^32^]

Pleasingly, the halide exchange reaction was also feasible using only catalytic amounts of Pd^I^ dimer **2** in the presence of an excess of ^*n*^Bu_4_NBr as a nucleophile (Figure 6). The proposed catalytic cycle involves an initial one‐ or two‐fold nucleophilic exchange of the μ‐bridging nucleophiles of the Pd^I^ dimer prior to oxidative addition of the aryl halide.[^31^, ^32^] The intermediately formed Pd^II^ dimers then undergo reductive elimination to yield the product and re‐form a Pd^I^ dimer. This new concept relies on the employed coupling partner (nucleophile) to overall be able to stabilize the dinuclear Pd^I^ framework. Notably, however, within this dinuclear catalysis concept the oxidative addition and transmetalation elementary steps are reversed, which circumvents the formation of potentially unreactive Pd^II^ by‐products during transmetalation (see Section 3.4). Moreover, since the nucleophilic exchange takes place at oxidation state (I) [rather than the usual (II) as in Pd^0^/Pd^II^ catalysis] different driving forces for the exchange process may overall exist, which in turn may enable privileged reactivities.

### 3.4 Distinct Bond‐Formations Harnessing Dinuclear Catalysis

Following the initial observation of dinuclear catalysis, our group examined the wider synthetic impact of this concept. Our developments focussed on using the iodide‐bridged Pd^I^ dimer **2** as a catalyst because this complex is completely air‐stable as a solid and can be stored on the bench without special precautions. The corresponding coupling partner was employed as a salt.

**Figure 7:**
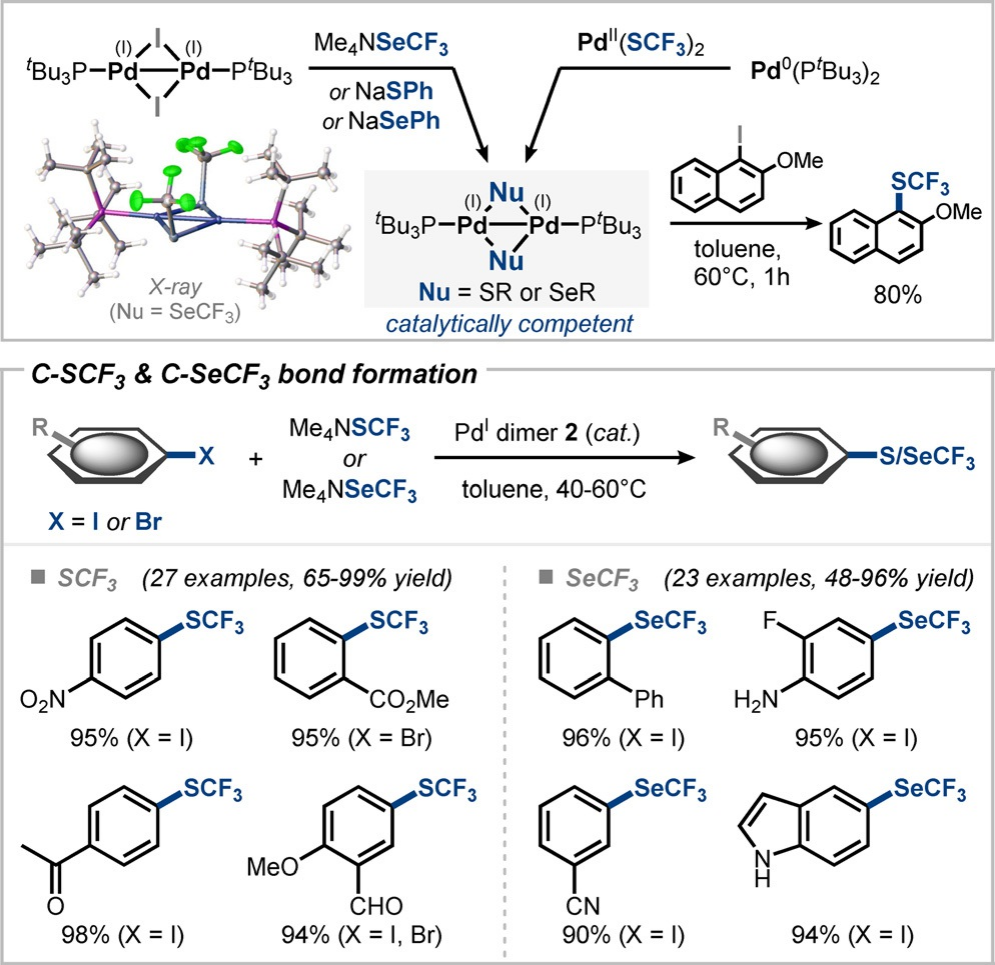
Application of dinuclear Pd^I^ catalysis in trifluoromethylthiolations and ‐selenolations: isolation of catalytically competent SCF_3_‐ and SeCF_3_‐bridged Pd^I^ dimers (top) and scope of the catalytic transformations (bottom).[^8a^, ^33a^]

We developed the direct catalytic trifluoromethylthiolation (C−SCF_3_ coupling) of a wide range of aryl iodides and bromides using the bench stable salt NMe_4_SCF_3_.[^8a^, ^33^] In this context, the Pd^I^ concept complemented existing Pd^0^‐ or Ni^0^‐based strategies that were limited in compatible aryl halide.^[34]^ The first direct catalytic C−SeCF_3_ bond formation of aryl iodides and bromides was also accomplished using the corresponding NMe_4_SeCF_3_ salt.^[8a]^ In both cases nucleophile exchange at the dinuclear Pd^I^ scaffold was confirmed in stoichiometric studies and the novel SCF_3_‐ and SeCF_3_‐bridged Pd^I^ dimers were isolated and characterized (Figure 7). Both complexes were also completely air‐stable. ^31^P and ^19^F NMR monitoring of their stoichiometric reaction with aryl iodide shows the step‐wise transfer of the trifluoromethylated chalcogenide nucleophiles from the Pd^I^ dimers to form the product. This is in line with the computed free‐energy pathway that suggests feasible activation barriers for the direct reactivity and an overall exergonic thermodynamic driving force. Using catalytic amounts of Pd^I^ dimer **2** along with chalcogenide nucleophile in the form of soluble tetramethylammonium salts Me_4_NSCF_3_ and Me_4_NSeCF_3_ ensured formation of the active dimers in situ and enabled catalytic turn‐over. The formed Pd^I^ dimers were found to be very robust and their recovery after the reaction was feasible by simple open‐atmosphere column chromatography and even allowed for their reuse in further reactions without significant loss in activity.[^8a^, ^33a^]

**Figure 8:**
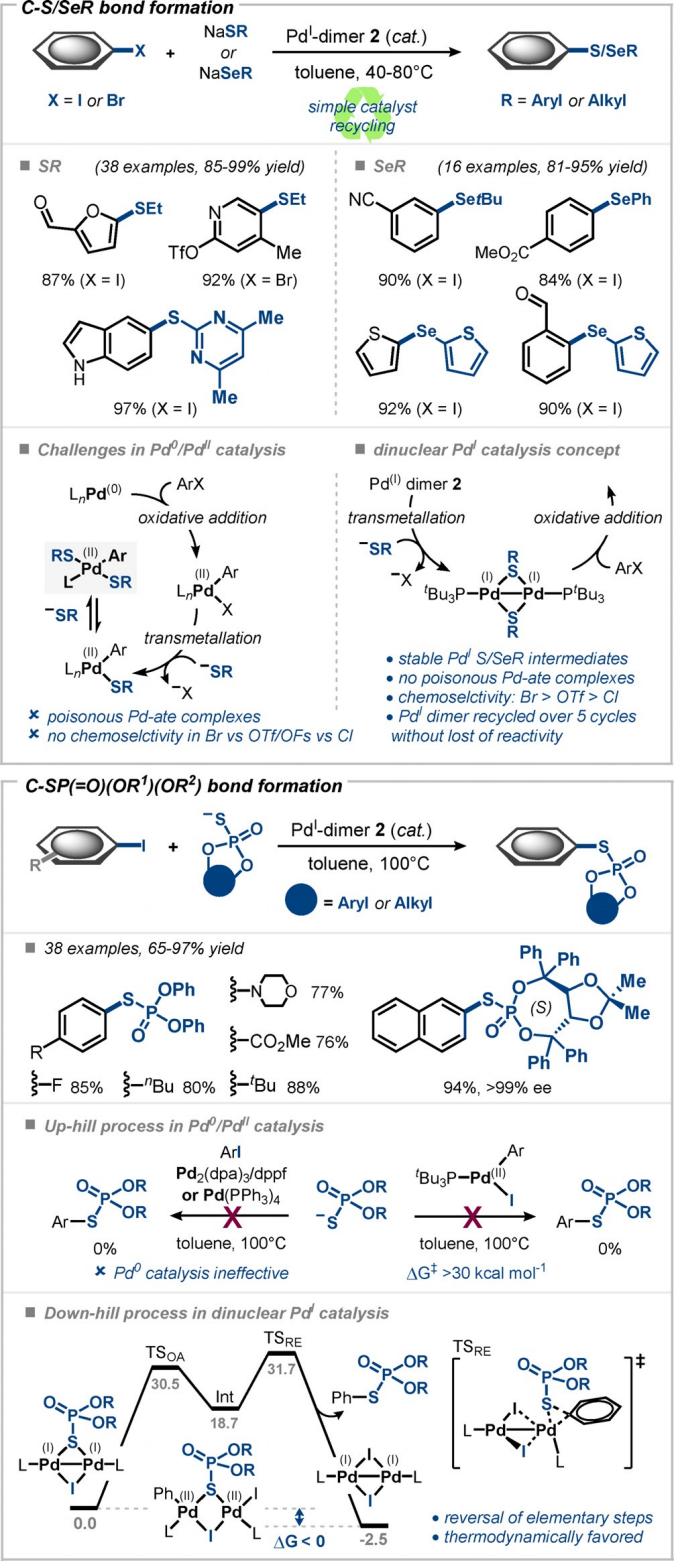
Heteroatom bond formation via dinuclear catalysis using thiolates or selenolates (top)^[35]^ and phosphorothioates (bottom).^[36]^

Following our initial work on the electron‐deficient SCF_3_/SeCF_3_ nucleophiles in Pd^I^ catalysis our group reported the thiolation and selenolation of aryl iodides and bromides using sodium thiolates and selenolates as electron‐rich nucleophilic coupling partners (Figure 8).^[35]^ In this case the dinuclear Pd^I^ catalysis concept provided advantages as compared to established Pd^0^/Pd^II^ chemistry. In particular, catalyst poisoning by the electron‐rich nucleophiles via formation of deactivated Pd^II^‐ate off‐cycle intermediates—a recognized problem in Pd^0^ catalysis—was not encountered. Moreover, the developed Pd^I^ protocols allowed for an exclusive and a priori predictable site‐selectivity as only aryl iodides and bromides were reactive in the presence of other potentially reactive functional groups such as C−Cl and C−OTf groups. For Pd^0^‐based strategies, site selectivity is often not general, but substrate‐specific and sometimes unpredictable, whereas for Pd^I^ it is dependent solely on the leaving group (see additional discussion below). Additionally, previously no catalytic methods existed for the direct selenolation of aryl halides. The remarkable stability and robustness of Pd^I^ dimer **2** allowed for several recovery cycles of the catalyst by simple column chromatography maintaining its catalytic activity.

Phosphorothioates (^−^SP(=O)(OR)_2_) were also demonstrated to be suitable coupling partners in Pd^I^‐mediated bond formation, which enabled the first C−SP(=O)(OR)_2_ coupling of aryl halides (Figure 8). By contrast, traditional Pd^0^/Pd^II^ catalysis failed to deliver the phosphorothioate cross‐coupling product as a consequence of a prohibitively high activation barrier for the reductive elimination from the key Pd^II^ intermediate and a lack of driving force.^[36]^ Stoichiometric experiments with Pd^I^ dimer **2** and ^31^P NMR analysis clearly indicated the formation of a new Pd^I^ dimer, demonstrating the competence of phosphorothioates to stabilize the Pd^I^ scaffold and the altered driving force for exchange at Pd^I^. Furthermore, computational analysis predicted a clear driving force for the direct reactivity of phosphorothioate‐bridged Pd^I^ dimers, which was confirmed in experiments. A number of aryl iodides were efficiently phosphorothioated using convenient tetramethylammonium phosphorothioate salts and the air‐stable Pd^I^ dimer **2** as catalyst. The protocol is general, operationally simple and tolerates structural diversity on the phosphorothioate backbone. Aryl esters as well as axially chiral phosphorothioates were coupled with high yields. The chiral information at phosphorus was retained in the coupling process (Figure 8).^[36]^

**Figure 9:**
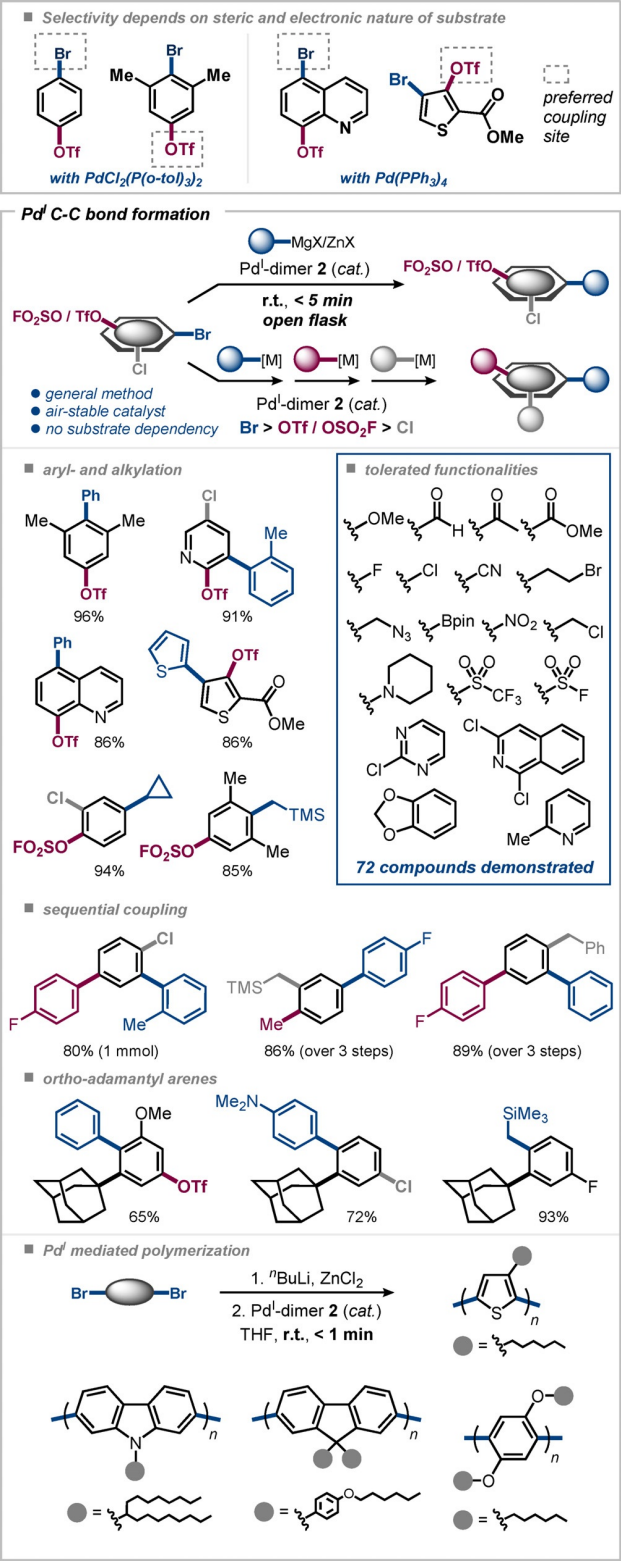
C−C bond formation by dinuclear Pd^I^ catalysis: site‐selective arylations and alkylations (top),[^22^, ^28^, ^39^, ^40^] modular, sequential diversification (middle),[^41^, ^42^] and polymerization (bottom).^[43]^

A long‐standing challenge in Pd^0^ catalysis is the site‐selective functionalization of poly(pseudo)halogenated arenes (bearing, for example, C−Br, C−Cl, C−OTf in the same molecule). The selectivity in standard Pd^0^/Pd^II^ catalysis is dependent on the catalyst/ligand, solvent, additives, and reaction conditions,[^8b^, ^37^] but most importantly, also the substrate itself.^[38]^ Subtle steric and electronic effects can vastly impact the selectivity. Consequently, selective coupling conditions identified for a given substrate may no longer give rise to selective coupling with the next. For instance, 4‐bromophenyl triflate underwent selective Kumada coupling at C−Br with PdCl_2_(P(*o*‐tol)_3_)_2_, but when two methyl groups were introduced *ortho* to C−Br a mixture of couplings at C−Br and C−OTf was observed under otherwise identical reaction conditions (Figure 9, top).[^28^, ^37i^] With Pd^I^ dimer **2** a fully a priori predictable arylation and alkylation protocol was achieved using organomagnesium^[22]^ or organozinc species as appropriate nucleophilic cross‐coupling partners.[^28^, ^39^] The method enables the functionalization at C−Br sites in the presence of other reactive functional groups, including C−OTf and/or C−Cl, within seconds to a few minutes at room temperature in air. The protocol also allows for larger‐scale applications. Both yield and selectivity were found to be fully substrate‐independent and even highly sterically demanding *ortho*‐adamantyl C−Br sites were selectively functionalized while leaving C−OTf and C−Cl sites untouched.^[40]^ Alkylation of aryl (pseudo)halides has been an especially challenging area of development. Challenges include β‐hydride eliminations as side processes or metal–halogen exchange. Few catalysts have been developed that efficiently overcame these challenges. Our tests of these state‐of‐the‐art alkylation catalysts gave no selectivity when challenged with poly(pseudo)halogenated arenes however. As such, the Pd^I^‐based C−C bond formations are a significant advance in being fully predictable in selectivity, air‐tolerant, and extremely efficient (reaction times of seconds to a few minutes).

While the Pd^I^ dimer **2** triggers solely C−Br functionalization in the presence of C−OTf or C−Cl in toluene or THF, in NMP, DMPU, DMI, or DMAc efficient functionalization of aryl triflates and chlorides can also be achieved.^[41]^ Culminated in the modular, site‐selective diversification of polyhalogenated arenes in the reactivity order Br>OTf>Cl, one‐pot double and triple functionalizations sequences were performed in less than 50 minutes under mild reaction conditions. The coupling procedure was further expanded to the functionalization of (or in the presence of) aryl fluorosulfates (Ar−OSO_2_F), a motif of increasing interest due its properties which are relevant to medicinal chemistry and material science, for which no site‐selective coupling had previously existed. The Pd^I^ dimer **2** enabled the site‐selective functionalization of C−Br sites in presence of the OSO_2_F (=OFs) groups as well as using the OFs moiety as a sustainable and inexpensive triflate surrogate.^[42]^

Furthermore, other C−C bond formations, such as the Heck reaction of terminal olefins and α‐arylations of esters and ketones, were also enabled by the air‐stable Pd^I^ dimer **2** showing the same exquisite chemoselectivity (I, Br≫Cl, OTf).^[44]^ Despite harsh conditions (100 °C in presence of base) recovery of the dinuclear Pd^I^ catalyst was possible after Heck reaction.^[44a]^ Bromide‐bridged dimer **1** had also been shown to be an efficient pre‐catalyst for α‐arylations, although specifically tailored conditions were required for different carbonyls.[^18a^, ^18d^, ^18e^, ^18f^] In contrast, using the air‐stable dimer **2** allowed for a more general method that enabled easy access to pharmaceutically relevant α‐arylated cyclopropyl ketones.^[44b]^

Our group further showed that the outstanding reactivity and selectivity of the Pd^I^ dimer **2** as cross‐coupling catalyst for small molecules remains viable as a general strategy to also polymerize valuable macromolecules, that is, polyfluorenes or poly‐*para*‐phenylenes.^[43]^ Starting from monomers containing two bromides, in situ lithiation and transmetalation to the corresponding organozinc enabled rapid polymerizations with good average molecular weights (*M*_n_) and polydispersities (PDI) using Pd^I^ dimer **2** as a catalyst, even in the presence of air. As compared to the single C−C bond formations above, the polymerization is similarly general, selective and rapid (in seconds) for a variety of different monomers and polymers. This is a significant advance to previous M^0/II^ polymerization strategies where for each monomer a specific catalyst needs to be tuned and usually long reaction times and/or elevated temperatures are required to achieve high conversion as well as the exclusion of oxygen is needed.^[45]^

## 4 Applications in Other Transformations

**Figure 10:**
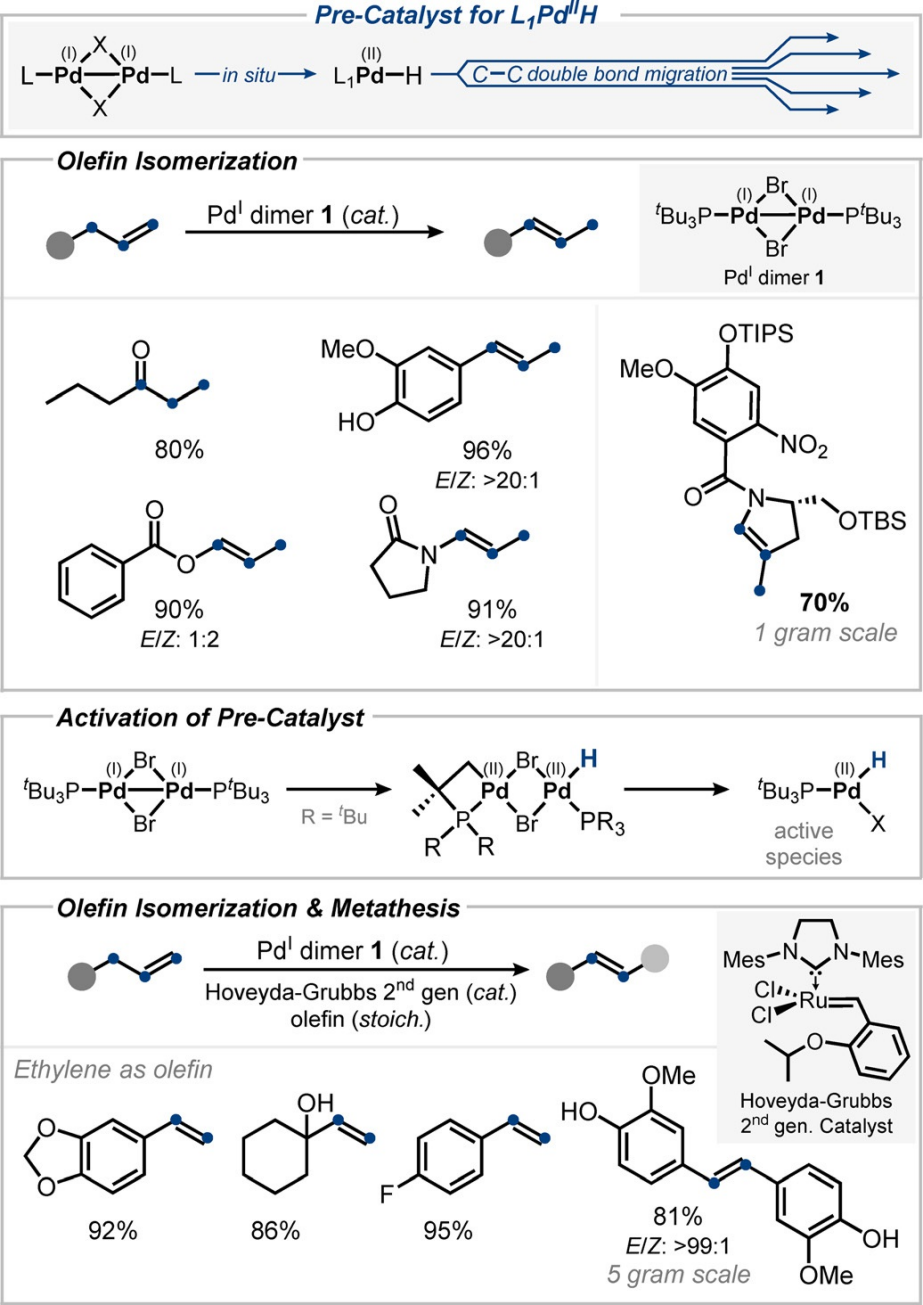
Isomerization (and metathesis) strategies with Pd^I^ dimer as pre‐catalyst.[^46^, ^48a^, ^48c^, ^49^]

Gooßen and co‐workers demonstrated the one‐bond olefin migration of allylic esters to make enol esters in moderate *E*/*Z* selectivity using [Pd(μ‐Br)(P^*t*^Bu_3_)]_2_ **1** (Figure 10).^[46]^ This as well as previous work[^7a^, ^27^] indicated that a [Pd^II^−H] is generated in situ from the labile dimer **1** under these conditions. Gooßen also found that since a monophosphine‐derived Pd^II^−H species is liberated, the corresponding isomerization can be coupled with metathesis in the same pot.^[47]^ Other typical isomerization catalysts, such as (P^*t*^Bu_3_)_2_Pd^II^(Cl)(H), release phosphine in situ, which in turn would inhibit the metathesis catalyst and therefore make them incompatible (Figure 10).^[48]^

Parker and co‐workers demonstrated the potential of this Pd^I^‐dimer‐catalyzed isomerization approach by implementing it as a key synthetic step in a total synthesis towards tesirine and pyrrolobenzodiazepines on a gram scale.^[49]^

The nature of the catalytically active Pd species—in particular its nuclearity as the key parameter determining its reactivity—was investigated in a preliminary DFT analysis in the original report^[46]^ and recently re‐investigated and ‐evaluated in a thorough DFT analysis in collaboration with the Koley group.^[50]^ Their DFT analysis ruled out that the Pd^I^ dimer itself is sufficiently activated to perform the isomerization of olefins. Instead, the data indicate that it serves as the source of a mononuclear Pd^II^−H species. The in situ release is suggested to proceed via cyclopalladation with one of the coordinating trialkyl phosphine ligands and eventually dissociation of the dimeric cyclopalladated Pd species to yield a mononuclear and highly reactive L_1_BrPd^II^−H.

The synthetic value of Pd^I^‐dimer‐catalyzed double‐bond migration and subsequent reactivity via a second catalytic transformation was furthermore demonstrated in the joint isomerization/hydroformylation of fatty acid methyl esters, reported by Vorholt and co‐workers in 2017.^[51]^

Although [Pd(μ‐Br)(P^*t*^Bu_3_)]_2_ **1** is, owing to its high reactivity, a potent pre‐catalyst in olefin isomerizations, it readily decomposes in solution when exposed to air.

**Figure 11:**
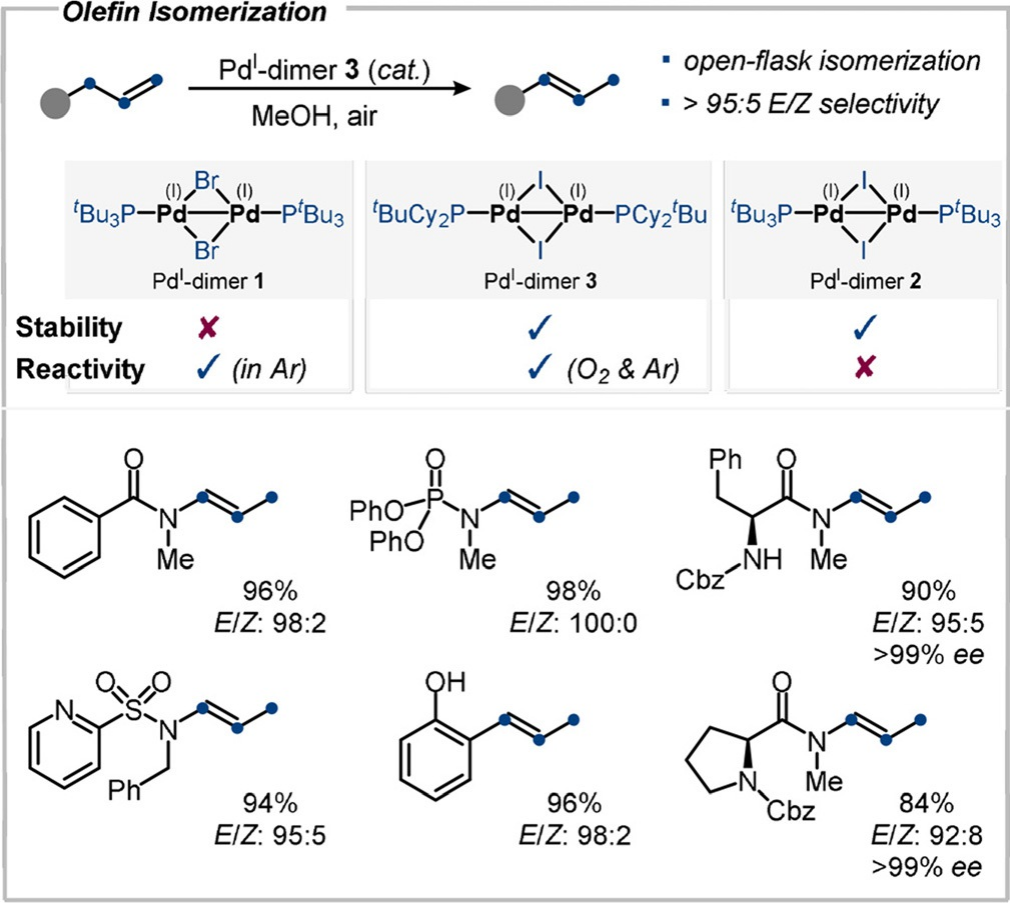
Isomerization with Pd^I^ dimer **3** as pre‐catalyst.^[8c]^

In 2020, our group established [Pd(μ‐I)(PCy_2_^*t*^Bu)]_2_ **3** as a new air‐stable dimer and extended the portfolio of Pd‐catalyzed olefin isomerizations (Figure 11).^[8c]^ Dimer **3** allows for a double‐bond isomerization for the first time under open‐flask conditions fully exposed to oxygen with more than 40 substrates in excellent yields and with high *E*/*Z* selectivities. The polar protic solvent MeOH proved to be optimal, which contrasts previous developments that relied on non‐polar solvents. By contrast, the bromide‐bridged Pd^I^ dimer **1** is ineffective under the same conditions as it is rapidly deactivated in the presence of oxygen. Conversely, the previous generation of air‐stable Pd^I^ dimers, that is, **2**, was not effective in this transformation either, as it is too robust and does not liberate Pd^II^−H. The reactions with **3** display a wide scope, leaving stereocenters in substrates untouched; amino acid derivatives were isomerized with high *E*/*Z* ratio. Furthermore, dimer **3** was also shown to be a potent catalyst for site‐selective C−C bond formations and even superior to **2** for C−OTf couplings that bear *ortho* substituents.

## 5 Summary and Outlook

In this Minireview we have highlighted the use of dinuclear Pd^I^ complexes as catalysts in a wide range of transformations. Their applications span from being efficient pre‐catalysts for highly active monophosphine Pd^0^ or Pd^II^−H species in cross‐coupling and olefin isomerization reactions, respectively, to direct dinuclear catalysis. While the air‐sensitive and labile bromide‐bridged Pd^I^ dimer [Pd^I^(μ‐Br)(P^*t*^Bu)_3_]_2_ received most attention in the earlier literature since 2002 for its success in triggering especially aminations or Suzuki cross‐couplings of aryl halides as well as α‐arylations of carbonyl compounds, the corresponding air‐stable iodide‐bridged Pd^I^ dimer [Pd^I^(μ‐I)(P^*t*^Bu)_3_]_2_ remained essentially unexplored in catalysis until a decade later. The latter is much more stable and frequently unreactive under standard cross‐coupling conditions, as it does not readily liberate Pd^0^. This feature has been harnessed since 2013 when the feasibility of dinuclear catalysis was established, both mechanistically as well as synthetically. Here, the Pd^I^−Pd^I^ bond stays intact during catalysis, which has enabled a range of selective transformations that are not amenable to Pd^0^/Pd^II^ catalytic cycles. Especially weaker nucleophiles could be implemented owing to the altered driving forces associated with exchanges at Pd^I^ as opposed to Pd^II^. The associated air‐stability of the Pd^I^ species allowed for its recovery and reuse (with multiple rounds of recycling). Moreover, as opposed to Pd^0^‐based catalysis, excellent a priori predictable selectivities for poly(pseudo)halogenated arenes, featuring aryl bromide, chloride, triflate, and/or fluorosulfate functionalities were achieved with [Pd(μ‐I)(P^*t*^Bu_3_)]_2_. Notably, the C−C bond formations were accomplished in seconds to a few minutes at room temperature under open‐flask conditions (tolerating oxygen). Given the exquisite selectivities paired with high reactivity, practicability (air tolerance, recyclability), as well as potential for diverse and privileged catalysis, we expect to see many more powerful applications in the years to come. Key in this area is to control the speciation of the Pd^I^ dimer, which in turn controls the mode of reactivity. As this is a delicate interplay of multiple factors, including ligands and additives, the area will continue to benefit greatly from fundamental insight to guide future developments and novel dimer generations.

## Conflict of interest

The authors declare no conflict of interest.

## Biographical Information

*Christoph Fricke received his BSc and his MSc degrees at the WWU Münster in Germany. He joined the Schoenebeck group at RWTH Aachen University for his PhD studies (2015–2019), where he was involved in homogeneous catalysis. In 2020, he joined the Gaunt group at the University of Cambridge for his postdoctoral studies*.

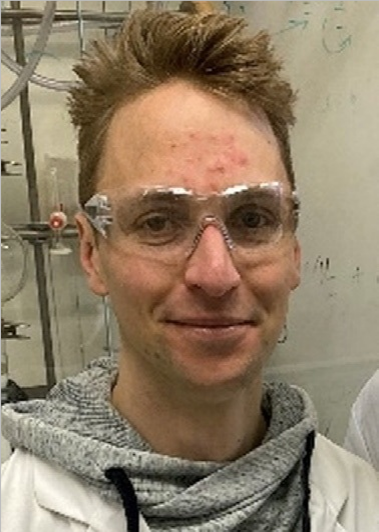

## Biographical Information

*Theresa Sperger conducted her BSc and MSc studies at the ETH Zürich in Switzerland. She joined the Schoenebeck group for her MSc thesis before undertaking her PhD from 2014–2018 in the same group, conducting combined experimental and computational studies on dinuclear Pd* ^*I*^ *complexes and their reactivities*.

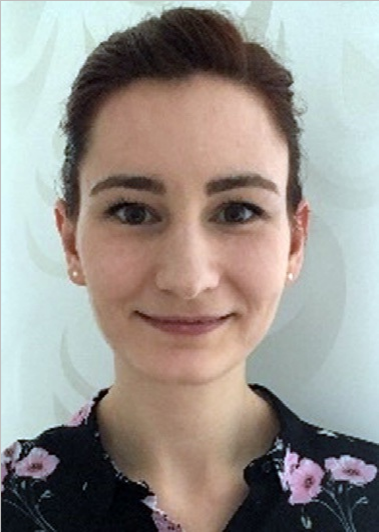

## Biographical Information

*Marvin Mendel undertook his BSc studies in chemistry at Ulm University. He moved to RWTH Aachen University for his MSc studies and joined the Schoenebeck group for his MSc thesis in the area of site‐selective Pd* ^*I*^ *catalysis before starting his PhD in May 2019 in the same group*.

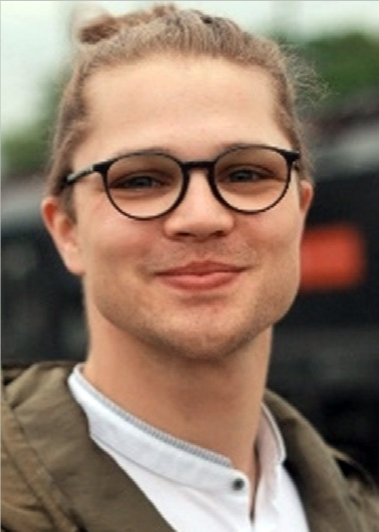

## Biographical Information

*Franziska Schoenebeck received her PhD in 2008 from the University of Strathclyde (Glasgow, UK) working with Prof. J. A. Murphy. After a postdoctoral stay with Prof. K. N. Houk at UCLA, she started her independent career at the ETH Zürich in 2010. In 2013 she was appointed Professor at the RWTH Aachen University, where she was promoted to Full Professor and Chair in 2016. Her research is based at the interface of synthetic organic, mechanistic, and computational chemistry with an emphasis in homogeneous metal catalysis*.